# Bactericidal Activity of Sodium Bituminosulfonate against *Staphylococcus aureus*

**DOI:** 10.3390/antibiotics11070896

**Published:** 2022-07-05

**Authors:** Elisa Heuser, Karsten Becker, Evgeny A. Idelevich

**Affiliations:** 1 Friedrich Loeffler-Institute of Medical Microbiology, University Medicine Greifswald, Ferdinand-Sauerbruch-Straße 1, 17489 Greifswald, Germany; elisa.heuser@med.uni-greifswald.de (E.H.); karsten.becker@med.uni-greifswald.de (K.B.); 2 Institute of Medical Microbiology, University Hospital Münster, Domagkstraße 10, 48149 Münster, Germany

**Keywords:** sodium bituminosulfonate, *Staphylococcus aureus*, bactericidal activity

## Abstract

Antibiotic resistance is increasing worldwide making it necessary to search for alternative antimicrobials. Sodium bituminosulfonate is a long-known substance, whose antimicrobial inhibitory activity has recently been re-evaluated. However, to the best of our knowledge, the bactericidal mode of action of this substance has not been systematically characterized. The aim of this study was to investigate the in vitro bactericidal activity of sodium bituminosulfonate by determining the minimal bactericidal concentrations (MBC), as well as the rapidity of bactericidal effect by time-kill curves. Clinical isolates of methicillin-susceptible (MSSA, *n* = 20) and methicillin-resistant (*mecA/mecC*-MRSA, *n* = 20) *Staphylococcus aureus* were used to determine MBC by a broth microdilution method. Sodium bituminosulfonate (Ichthyol^®^ light) was tested in double-dilution concentration steps ranging from 0.03 g/L to 256 g/L. For time-kill analysis, two reference and two clinical *S. aureus* strains were tested with different concentrations of sodium bituminosulfonate (1× minimal inhibitory concentration (MIC), 2× MIC, 4× MIC, 16× MIC and 256× MIC). For MSSA isolates, MBC_50_, MBC_90_ and the MBC range were 0.5 g/L, 1.0 g/L and 0.125–1.0 g/L; (MBC/MIC ratio)_50_, (MBC/MIC ratio)_90_ and the range of the MBC/MIC ratio were 4, 4 and 1–8, respectively. Among MRSA isolates, MBC_50_, MBC_90_ and the MBC range amounted to 0.5 g/L, 1.0 g/L and 0.06–1.0 g/L; (MBC/MIC ratio)_50_, (MBC/MIC ratio)_90_ and the range of the MBC/MIC ratio were 2, 4 and 1–8, respectively. Time-kill kinetics revealed a bactericidal effect after 30 min for sodium bituminosulfonate concentrations of 16× MIC and 256× MIC. The bactericidal activity against MSSA and MRSA was demonstrated for sodium bituminosulfonate. The killing was very rapid with the initial population reduced by 99.9% after only short incubation with concentrations of 16× MIC and higher.

## 1. Introduction

The burden of bacterial resistance against antibiotics is steadily increasing [1,2]. The development of antimicrobial resistance (AMR) is driven by use of antibiotics in humans, animals and the environment, as well as the worldwide spread of resistant bacteria [3]. Although considerable improvements in the control of methicillin-resistant *Staphylococcus aureus* (MRSA) have been made in certain countries, in the WHO priority list of antibiotic-resistant bacteria, MRSA is still ranked as a high priority pathogen [4]. In 2019, MRSA caused more than 100,000 deaths and 3.5 million disability-adjusted life-years attributable to AMR [5]. Despite some progress achieved in the antibacterial pipeline in recent years, the pipeline outlook remains unfavorable [6,7]. In the context of a scarcity of new antimicrobial treatments, the re-examination of old substances with antimicrobial activity has potential value [8]. Such re-evaluation includes the collection of in vitro susceptibility data as well as consideration of clinical evidence, including pharmacokinetic/pharmacodynamic analyses [9,10]. Sodium bituminosulfonate, a long-known substance derived from sulfur-rich oil shale and commonly known as “Ichthyol^®^, light” [11], has been used to treat various conditions, particularly in dermatology, including skin infections, for nearly a century [12,13,14]. The in vitro antimicrobial activity of sodium bituminosulfonate has recently been re-evaluated according to current international guidelines on antimicrobial susceptibility testing (AST) [15]. To the best of our knowledge the first time this has been undertaken, we performed a systematic investigation and characterization of the bactericidal activity of sodium bituminosulfonate against *S. aureus*.

## 2. Results

### 2.1. Determination of MIC and MBC

Of 20 clinical MRSA strains, the *mecA* gene was detected in 19 strains, whereas one strain possessed the *mecC* gene. All MSSA isolates tested *mecA*/*mecC*-negative. For MSSA as well as for MRSA clinical isolates, the MIC_50_ and MIC_90_ values were 0.125 g/L and 0.25 g/L, respectively. The MIC range was 0.06–0.5 g/L for MSSA and 0.06–0.25 g/L for MRSA. The MBC_50_ and MBC_90_ values were equal for MSSA and MRSA and amounted to 0.5 g/L and 1.0 g/L, respectively (Table 1). MBC ranges were 0.125–1.0 g/L and 0.06–1.0 g/L for MSSA and MRSA, respectively. The (MBC/MIC ratio)_50_, (MBC/MIC ratio)_90_ and the range of the MBC/MIC ratio are shown in Table 1.

The MICs of vancomycin determined for *S. aureus* ATCC 29213 by general QC testing were within the ranges recommended by EUCAST and CLSI [15,16].

### 2.2. Time-Kill Curves

**Figure 1:**
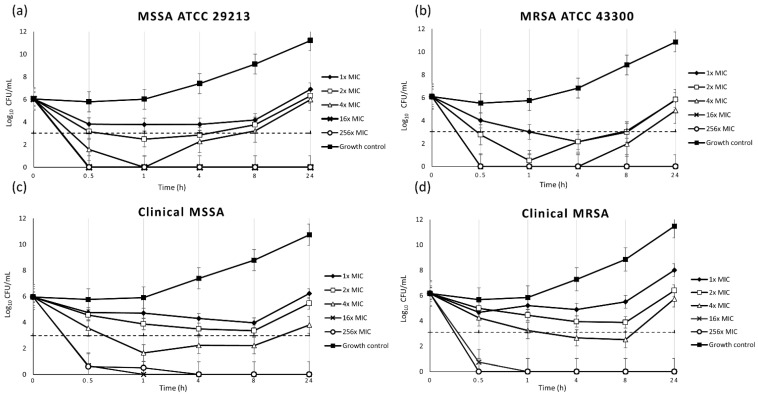
Time-kill curves for sodium bituminosulfonate against two reference strains (**a**,**b**) and two clinical strains (**c**,**d**) of *Staphylococcus aureus*. The threshold (dashed line) denotes 99.9% decreases in CFU/mL. MSSA, methicillin-susceptible *S. aureus*; MRSA, methicillin-resistant *S. aureus*; MIC, minimal inhibitory concentration.

Time-kill kinetic investigations demonstrated a rapid bactericidal effect of sodium bituminosulfonate against MSSA and MRSA. Confirmation of the initial inoculum revealed a mean real bacterial density of 1.1 × 10^6^ CFU/mL and 1.3 × 10^6^ CFU/mL for the reference strains MSSA ATCC 29213 and MRSA ATCC 43300, respectively, 1 × 10^6^ for clinical MSSA and 1.5 × 10^6^ for clinical MRSA strains. At concentrations of 16 × MIC and 256 × MIC, a bactericidal efficacy of 99.9% inoculum reduction by sodium bituminosulfonate was already achieved after 30 min of incubation. Regrowth was observed at 1 × MIC, 2 × MIC and 4 × MIC within 4 to 24 h, whereas killing without regrowth was documented at 16 × MIC and 256 × MIC (Figure 1).

## 3. Discussion

*S. aureus* is one of the most common and important pathogens that can cause infections of skin and soft tissues, which are particularly difficult to treat when originating from methicillin-resistant strains [17]. Methicillin resistance in *S. aureus* is mediated by the *mecA, mecB* or *mecC* genes and leads to resistance to almost all β-lactam antibiotics [18,19,20,21]. Alternative antimicrobial substances may be important for the successful treatment of such infections or for the eradication of cutaneous colonization by *S. aureus*. Bituminosulfonate compounds have been known for over 100 years [22,23]. Data obtained from older studies—performed with various derivatives and formulations—showed good antimicrobial effects against Gram-positive bacteria, such as staphylococci and streptococci [24,25,26]. The activity of sodium bituminosulfonate against Gram-positive bacteria has recently been confirmed in an in vitro study [27]. Notably, another very recent study found only a low potential for resistance development in *S. aureus* after exposure to this derivate [28]. For comparison purposes, the antimicrobial inhibitory effect of sodium bituminosulfonate against *S. aureus* was also demonstrated in our study (Table 1). Its MIC values against 40 clinical and two reference *S. aureus* strains were found to be below the concentrations contained in commercially available preparations and the results were comparable to previous results [25,26,27,28]. The bactericidal efficacy of sodium bituminosulfonate was demonstrated by MBC determination. In agreement with international guidelines, a 99.9% killing of bacteria was considered as the criterion for a bactericidal effect [29]. To the best of our knowledge, no data are available on the rapidity of the bactericidal effect of sodium bituminosulfonate. Therefore, we characterized the bactericidal effect of sodium bituminosulfonate using a time-kill methodology [29,30]. In this first study of sodium bituminosulfonate’s bactericidity, a very rapid bactericidal effect against MSSA and MRSA was observed, with 99.9% of the inoculum already killed after a short incubation time of 30 min by concentrations corresponding to 16× MIC and 256× MIC (Figure 1). Regrowth—a phenomenon occasionally observed with the time-kill methodology [31,32]—was noted at lower concentrations of 1× MIC, 2× MIC and 4× MIC, while the higher concentrations of 16× MIC and 256× MIC caused irreversible killing. In conclusion, a bactericidal effect was demonstrated for sodium bituminosulfonate by MBC determination on a clinical collection of MSSA and MRSA strains. Detailed analyses of time-kill kinetics revealed very rapid bactericidal activity. This study contributes to the deeper in vitro characterization of the antimicrobial effects of sodium bituminosulfonate by investigating MBCs and time-kill curves under standardized conditions and has confirmed sodium bituminosulfonate as a promising alternative to commonly used topical antibiotics.

## 4. Materials and Methods

### 4.1. Bacterial Strains and Antimicrobial Substance

For the determination of minimal inhibitory concentrations (MICs) and minimal bactericidal concentrations (MBCs), 40 clinical isolates of *S. aureus* from routine diagnostics of the Friedrich Loeffler-Institute of Medical Microbiology, University Medicine Greifswald, in 2019–2021 were used, including 20 consecutive MSSA isolates and 20 consecutive MRSA isolates. The isolates were recovered from blood, urine, wounds, aspirates and respiratory samples. Only one isolate per patient was eligible. For time-kill experiments, two of these clinical strains (one MSSA and one MRSA) were used, as well as two reference strains (MSSA ATCC 29213 and MRSA ATCC 43300). The presence of *mec* genes in MRSA strains was confirmed using a loop-mediated isothermal amplification assay (eazyplex^®^ MRSAplus, Amplex Diagnostics, Gars-Bahnhof, Germany) according to the user’s manual [33].

Sodium bituminosulfonate (Ichthyol^®^ light) was provided by the Ichthyol-Gesellschaft Cordes, Hermanni & Co. (Hamburg, Germany).

### 4.2. Determination of MIC and MBC

For MIC determination, a broth microdilution method was used according to the recommendations of the Clinical and Laboratory Standards Institute (CLSI) and the International Organization for Standardization (ISO) [15,34,35]. The strains were cultivated overnight on Columbia blood agar plates at 35 °C. Afterwards, colony material from the overnight culture was adjusted to the turbidity standard of McFarland 0.5 in 0.9% saline. The cultures were diluted in cation-adjusted Mueller–Hinton broth (CA-MHB, BD Diagnostics, Heidelberg, Germany) to obtain a final test inoculum of 5 × 10^5^ CFU/mL. The testing was performed in sterile U-bottom microtiter plates (Brand, Wertheim, Germany) with an incubation time of 18 ± 2 h at 35 °C in ambient air. Sodium bituminosulfonate was tested in double-dilution concentration steps ranging from 0.03 g/L to 256 g/L. As no quality control (QC) ranges exist for the susceptibility testing of sodium bituminosulfonate, MIC determination was additionally performed for *S. aureus* ATCC 29213 with vancomycin to control for the overall performance of the testing procedures.

For the determination of MBCs, 10 µL samples from the clear wells were sub-cultured on tryptic soy agar plates (TSA, BD Diagnostics, Heidelberg, Germany) and colonies were counted after overnight incubation at 35 °C. The minimal concentration needed to kill at least 99.9% of the initial inoculum was considered as the MBC, according to CLSI [29]. All MIC and MBC determinations were performed in triplicate and median values were calculated for analysis.

### 4.3. Time-Kill Curves

To determine the rapidity of the bactericidal effect of sodium bituminosulfonate against *S. aureus*, a time-kill kinetic methodology was used. The initial liquid culture prepared in 10 mL tryptic soy broth (TSB) from overnight growth on Columbia blood agar was incubated for 3 h at 35 °C and 160 rpm. After adjusting to the 0.5 McFarland turbidity standard and dilution, 5 mL of a suspension containing approximately 10^6^ CFU/mL was transferred into glass flasks with different concentrations of sodium bituminosulfonate (1 × MIC, 2 × MIC, 4 × MIC, 16 × MIC and 256 × MIC for the respective isolate). The suspensions were incubated at 35 °C and 160 rpm for 0.5 h, 1 h, 4 h, 8 h and 24 h. After incubation, 200-µL samples were collected and serially diluted, followed by plating of 10 µL in triplicate on TSA plates. After overnight incubation at 35 °C, the colonies were counted, and average values were calculated. A growth control without antimicrobial substance, as well as a sterile control, were used in each experiment. The time-kill curves were performed in triplicate.

## Figures and Tables

**Table 1:** Bactericidal activity of sodium bituminosulfonate against methicillin-susceptible (MSSA, *n* = 20) and methicillin-resistant (MRSA, *n* = 20) *Staphylococcus aureus* isolates.

Strains	MIC_50_ (g/L)	MIC_90_ (g/L)	MIC Range (g/L)	MBC_50_ (g/L)	MBC_90_ (g/L)	MBC Range (g/L)	(MBC/MIC Ratio)_50_	(MBC/MIC Ratio)_90_	(MBC/MIC Ratio) Range
MSSA	0.125	0.25	0.06–0.5	0.5	1.0	0.125–1.0	4	4	1–8
MRSA	0.125	0.25	0.06–0.25	0.5	1.0	0.06–1.0	2	4	1–8

## Data Availability

The data for the study are available in the article.
